# Tal2c Activates the Expression of *OsF3H_04g_* to Promote Infection as a Redundant TALE of Tal2b in *Xanthomonas oryzae* pv. *oryzicola*

**DOI:** 10.3390/ijms222413628

**Published:** 2021-12-20

**Authors:** Tao Wu, Haimiao Zhang, Yunya Bi, Yue Yu, Haifeng Liu, Hong Yang, Bin Yuan, Xinhua Ding, Zhaohui Chu

**Affiliations:** 1 State Key Laboratory of Crop Biology, Shandong Agricultural University, Tai’an 271018, China; wtluochen@sina.com (T.W.); zhanghhhmiao@163.com (H.Z.); sdauzbyy@163.com (Y.Y.); hliu1987@sdau.edu.cn (H.L.); 2 State Key Laboratory of Hybrid Rice, Hubei Hongshan Laboratory, College of Life Sciences, Wuhan University, Wuhan 430072, China; yunyabi@163.com; 3 Shandong Provincial Key Laboratory for Biology of Vegetable Diseases and Insect Pests, College of Plant Protection, Shandong Agricultural University, Tai’an 271018, China; 4 State Key Laboratory of Biocatalysis and Enzyme Engineering, School of Life Sciences, Hubei University, Wuhan 430062, China; yang@hubu.edu.cn; 5 Institute of Plant Protection and Soil Fertilizer, Hubei Academy of Agricultural Sciences, Wuhan 430064, China; yuanbin2000@139.com

**Keywords:** rice, bacterial leaf streak, disease resistance, TALEs, 2OGD, *Xanthomonas oryzae*

## Abstract

*Xanthomonas oryzae* delivers transcription activator-like effectors (TALEs) into plant cells to facilitate infection. Following economic principles, the redundant TALEs are rarely identified in Xanthomonas. Previously, we identified the Tal2b, which activates the expression of the rice 2-oxoglutarate-dependent dioxygenase gene *OsF3H_03g_* to promote infection in the highly virulent strain of *X. oryzae* pv. *oryzicola* HGA4. Here, we reveal that another clustered TALE, Tal2c, also functioned as a virulence factor to target rice *OsF3H_04g_*, a homologue of *OsF3H_03g_*. Transferring Tal2c into RS105 induced expression of *OsF3H_04g_* to coincide with increased susceptibility in rice. Overexpressing *OsF3H_04g_* caused higher susceptibility and less salicylic acid (SA) production compared to wild-type plants. Moreover, CRISPR–Cas9 system-mediated editing of the effector-binding element in the promoters of *OsF3H_03g_* or *OsF3H_04g_* was found to specifically enhance resistance to Tal2b- or Tal2c-transferring strains, but had no effect on resistance to either RS105 or HGA4. Furthermore, transcriptome analysis revealed that several reported SA-related and defense-related genes commonly altered expression in *OsF3H_04g_* overexpression line compared with those identified in *OsF3H_03g_* overexpression line. Overall, our results reveal a functional redundancy mechanism of pathogenic virulence in Xoc in which tandem Tal2b and Tal2c specifically target homologues of host genes to interfere with rice immunity by reducing SA.

## 1. Introduction

During rice production, the Xanthomonas phytopathogen *Xanthomonas oryzae* pv. *oryzae* (Xoo) and *Xanthomonas oryzae* pv. *oryzicola* (Xoc) cause bacterial blight (BB) and bacterial leaf streak (BLS) diseases and yield losses of up to 50% and 32% under favorable conditions, respectively [1,2]. Currently, BLS has attracted more attention because of its increasing risk of frequent outbreaks and tremendous damage in Asia and Africa [3,4]. In China, BLS has also attracted attention because it is a plant quarantine disease for restricted seed production and commercial sales [5]. In addition to other *Xanthomonas* species, Xoc mainly uses the bacterial type III secretion system to deliver dozens of type III effectors, including transcription activator-like effectors (TALEs) and non-TALE effectors (non-TALEs), into rice to serve as virulence or avirulence factors [1]. TALEs belong to a unique family of transcription activators that conservatively consist of N-terminal, nuclear localization signal (NLS) motifs and acidic transcriptional activation domains (ADs) in the C-terminus and tandem repeats composed of 33 to 35 amino acids in the central region [6]. Each central repeat contains two hypervariable residues termed repeat-variable diresidue (RVD) at positions 12 and 13 that determine the recognition of one base in the DNA [7]. In a sequential fashion, each TALE contains up to 33.5 tandem repeats, which determine the specific recognition DNA sequence named the effector-binding elements (EBEs) on target genes [7,8]. After translocation into host nuclei, TALEs usually mimic the plant transcription factors to activate the expression of target genes.

Several non-TALEs have biological virulence functions in Xoc. For example, XopAJ/AvrRxo1 functions as an NAD kinase to suppress ROS burst [9,10] and XopC functions as an atypical kinase that phosphorylates OSK1 to suppress stomatal immunity [11]. Most candidate targeted genes for each TALE in sequenced Xoc strains have been predicted [12,13]. The development of functional analysis of TALEs is proceeding rapidly in Xoc. For instance, Tal2g was the first identified virulent TALE in Xoc BLS256, and it targets EBE in the promoter and activates the expression of the rice sulfate transporter gene.

*OsSULTR3;6* promotes infection [13]. Tal2h is a truncated TALE in BLS256 that interacts with and inhibits *Xo-1*-mediated resistance in Carolina Gold Select rice independent of DNA-binding activity [14,15]. Tal7 targets rice cyclin-D4-1 and GATA zinc finger family protein genes to repress *avrXa7/Xa7*-mediated defense in rice [16]. However, overexpression of Tal2a in BLS256 decreased virulence by targeting *UCH*, a rice ubiquitin carboxy-terminal hydrolase gene [17]. Tal2b (also known as TalBR1 and TalAQ3/Tal9b in Xoc and Xoo, respectively) and Tal2c (TalBL1) are clustered in some Xoc strains, such as BLS256 and HGA4 [4]. They were predicted to target genes encoding rice 2-oxoglutarate-dependent dioxygenase (2OGD), LOC_Os03g03034 (*OsF3H_03g_*) and LOC_Os04g49194 (*OsF3H_04g_*) [18]. Interestingly, a single mutant of Tal2b or Tal2c did not affect virulence in BLS256 [13], suggesting that they may present redundant functions with each other. Recently, we identified that Tal2b could enhance the pathogenicity of RS105 by targeting *OsF3H_03g_* to inactivate salicylic acid (SA) [4]. However, the redundancy function of Tal2c and its putative target *OsF3H_04g_* remain unclear.

The 2OGD family is involved in oxidative and hydroxylated reactions of different kinds of plant metabolites, and it is classified into four categories: DOXA, DOXB, DOXC and JMJ [19,20]. The DOXA and DXOB families are usually involved in DNA demethylation and proline hydroxylation, respectively. The JMJ family usually functions in histone lysine residue demethylation. The DOXC family may participate in the metabolism of various hormones and secondary metabolites [21], such as hydroxylation of SA, one of the key plant regulators involved in various plant defense responses [22]. In Arabidopsis, *DMR6/S5H* produces the inactive form of 2,5-dihydroxybenzoic acid (2,5-DHBA) from the substrate of SA. A mutant of DMR6/S5H could result in high accumulation of SA and resistance to *Hyaloperonospora parasitica* [23,24,25]. The DMR6/S5H homologue, S3H/DLO1, functions in the process of 2,3-dihydroxybenzoic acid (2,3-DHBA) by hydroxylating SA in vivo [26]. Recently, the loss of function of DMR-like genes was found to confer resistance to different phytopathogens in several plant species. For instance, gene editing of *StDMR6-1* in potato, *ObDMR6* in sweet basil and *MusaDMR6* in banana was found to specifically enhance resistance to late blight [27], downy mildew [28] and banana *Xanthomonas* wilt [29]. Moreover, gene editing of *SlDMR6-1* increases broad-spectrum disease resistance to bacteria, oomycetes and fungi in tomato [30]. In rice, the flavanone 3-hydroxylase (F3H) gene *OsF3H_03g_* is the target of Tal2b and Tal9b from Xoc and Xoo, respectively [13,18], which is involved in positively regulating resistance to brown planthoppers by accumulating flavonoid content [31], as well as negatively regulating resistance to BB, BLS and sheath blight along with a reduction of SA [4].

The CRISPR–Cas9 system is a powerful tool for studying the function of genes in many organisms [32]. Currently, it has been successfully applied for editing the promoter EBE of several susceptibility genes to generate broad-spectrum resistance to BB and BLS in rice. The sucrose transporter genes *OsSWEET11*, *OsSWEET13* and *OsSWEET14* are specific targets of the Xoo TALEs PthXo1, PthXo2 and PthXo3/AvrXa7, and they confer broad-spectrum resistance to BB after EBE edition in rice [33,34,35]. The EBE edited lines in the promoter of *OsSULTR3;6* increase resistance to Xoc strains BLS256 and RS105 [36]. Furthermore, gene editing of all three EBEs in the promoters of *OsSWEET11*, *OsSWEET14* and *OsSULTR3;6* confers resistance to both BLS and BB without any effect on agronomic traits in rice [37]. Thus, discovery of the recognition of TALEs and susceptible genes provides important guidance to generate resistant rice by editing EBEs.

Recently, we identified a highly virulent strain of Xoc HGA4 that showed four expanded TALEs not found in RS105. We found that Tal2b increases the virulence of RS105 by activating the expression of *OsF3H_03g_* [4]. The main objective of this study was to investigate the virulence contributions for other TALEs. Aimed at this goal, we focused on Tal2c, which is one of the four expanded TALEs that also act as a virulence factor to increase pathogenicity after introduction into RS105. Furthermore, we overexpressed (OE) the targeted gene of *OsF3H_04g_*, edited EBE editing in the *OsF3H_04g_* or *OsF3H_03g_* promoter and performed transcriptome analysis of the *OsF3H_04g_* OE line. All results suggest that the pair of TALEs, Tal2b and Tal2c, target two 2OGD family genes to redundantly regulate susceptibility to BLS in rice.

## 2. Results

### 2.1. Tal2c Acts as a Virulence Factor

**Figure 1:**
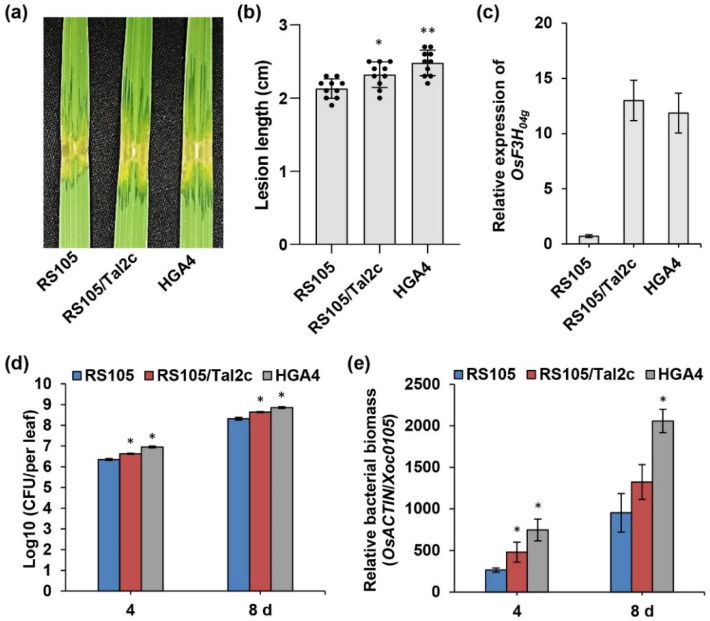
Tal2c from the Xoc strain HGA4 enhances the pathogenicity of Xoc strain RS105 by increasing the expression of *OsF3H_04g_*. (**a**) Image of lesion expansion and (**b**) lesion length in ZH11 rice leaves after inoculation with RS105, RS105/Tal2c and HGA4 at 14 days. Data represent the means ± SD, *n* = 10. Asterisks represent significant differences between RS105 and RS105/Tal2c, RS105 and HGA4 (* *p* ≤ 0.05, ** *p* ≤ 0.01, Student’s *t*-test). (**c**) Relative expression of *OsF3H_04g_* at 4 days post inoculation (dpi) with RS105, RS105/Tal2c and HGA4. The internal control was used, *OsACTIN*. Data represent the means ± SD, *n* = 3. (**d**) Bacterial populations and (**e**) relative bacterial biomass of RS105, RS105/Tal2c and HGA4 in ZH11 rice at 4 and 8 days after inoculation. Data represent the means ± SD, *n* = 3. Total genomic DNA of ZH11 rice inoculated with Xoc strains at 0 dpi was used as a control. Asterisks represent significant differences between RS105 and RS105/Tal2c, RS105 and HGA4 (* *p* ≤ 0.05, Student’s *t*-test).

There are four expanded TALEs in Xoc HGA4 but not in RS105, namely Tal2b, Tal2c, Tal2d and Tal2e [4]. Single mutants of Tal2b or Tal2c had no significant effect on the virulence of BLS256 [13]. Tal2b has been identified as a virulence factor for enhanced susceptibility after introduction into RS10 [4]. Thus, we further introduced Tal2c into RS105 for pathogenicity investigation. As shown in Figure 1a,b, RS105/Tal2c (2.32 ± 0.17 cm) caused an intermediate lesion length longer than that of RS105 (2.13 ± 0.13 cm) but less than that of HGA4 (2.48 ± 0.17 cm). Compared with RS105, both RS105/Tal2c and HGA4 induced an increase in *OsF3H_04g_* expression by 10-fold at 4 days post inoculation (Figure 1c). Consistent with the phenotype, we also observed that the bacterial population and relative biomass of RS105/Tal2b were larger than those of RS105 but lower than those of HGA4 in rice leaves during infection (Figure 1d,e). These results suggest that Tal2c acts as a virulence factor in Xoc HGA4.

### 2.2. Tal2c Targets OsF3H_04g_ and Tandem Pairs of Tal2b in Xoc

Tal2c was predicted to bind the EBE sequence at the *OsF3H_04g_* promoter region and activate the expression of *uidA* driven under the promoter of *OsF3H_04g_* in *N. benthamiana* using a transient expression system [13,18]. We also found that both Tal2c-containing strains of RS105/Tal2c and HGA4 could activate the expression of *OsF3H_04g_* (Figure 1c). To further validate the activation ability of Tal2c, we coexpressed it with green fluorescence protein (GFP) driven under the promoter of *OsF3H_04g_* (04gPRO-GFP) or EBE deletion (04gPRO∆EBE-GFP). Compared to each control, coexpressed Tal2c activated strong fluorescence with 04gPRO-GFP but not with 04gPRO∆EBE-GFP (Figure S1). There are different names for homologues to Tal2c in sequenced Xoc strains (Figure S2). Based on the characteristic pair of RVDs at positions 12 and 13 of each central repeat, Tal2c orthologues from different Xoc strains were annotated according to a previous report [38]. Interestingly, we found that Tal2c appeared in tandem with Tal2b in most sequenced Xoc strains (Table 1).

### 2.3. Overexpression of OsF3H_04g_ Increases Susceptibility to Xoc in Rice

**Figure 2:**
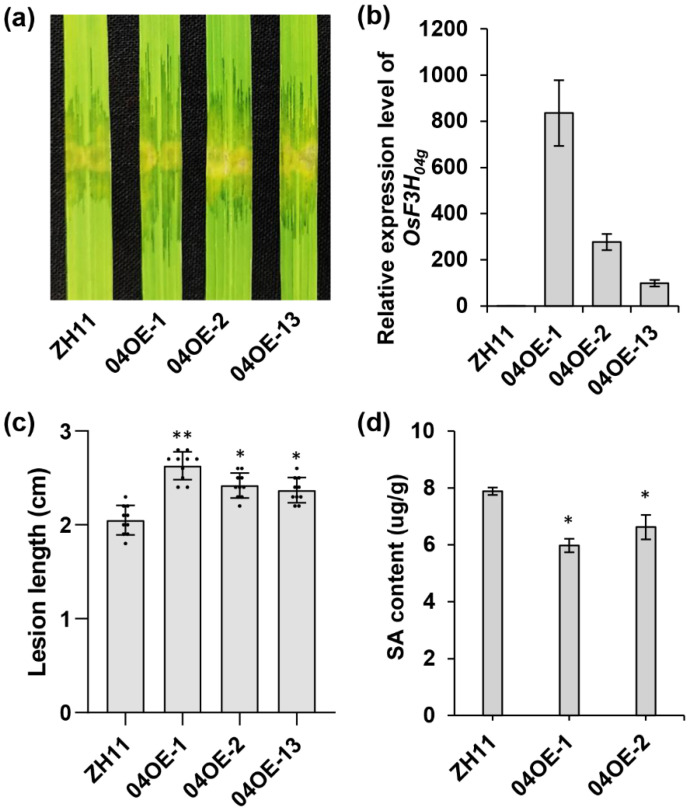
Overexpression of *OsF3H_04g_* increases rice susceptibility to Xoc strain RS105. (**a**) Photograph of lesion expansion at 14 dpi with RS105 in ZH11 and *OsF3H_04g_* OE lines. (**b**) Quantification of *OsF3H_04g_* expression in the ZH11 and *OsF3H_04g_* overexpression (OE) lines (04OE-1, 04OE-2 and 04OE-13). *OsACTIN* was used as an internal control. Data represent the means ± SD, *n* = 3. (**c**) Diagram of lesion lengths in ZH11 and *OsF3H_04g_* OE lines at 14 dpi with RS105. Data represent the means ± SD, *n* = 10. Asterisks represent significant differences between ZH11 and *OsF3H_04g_* OE lines (* *p* ≤ 0.05, ** *p* ≤ 0.01, Student’s *t*-test). (**d**) Salicylic acid (SA) content in ZH11 and *OsF3H_04g_* OE lines (04OE-1 and 04OE-2). Data represent the means ± SD, *n* = 3. Asterisks represent significant differences between ZH11 and *OsF3H_04g_* OE lines (* *p* ≤ 0.05, Student’s *t*-test).

Tal2c enhances the pathogenicity of RS105 and activates the expression of *OsF3H_04g_* in rice (Figure 1a,c). To investigate the function of *OsF3H_04g_*, we generated 15 *OsF3H_04g_* overexpression (OE) lines driven under the ubiquitin promoter in ZH11. Three lines named 04OE-1, 04OE-2 and 04OE-13 were selected for the detection of *OsF3H_04g_* expression in the T_2_ generation (Figure 2a). Compared with wild-type ZH11, the expression of *OsF3H_04g_* was specifically increased 834-, 276- and 98-fold in the 04OE-1, 04OE-2 and 04OE-13 lines (Figure 2b). Additionally, disease resistance assays were performed on three OE lines, and they caused more severe symptoms in all *OsF3H_04g_* OE lines than in ZH11 after inoculation with RS105 (Figure 2a). The lesion lengths of 04OE-1 (2.63 ± 0.15 cm), 04OE-2 (2.42 ± 0.13 cm) and 04OE-13 (2.37 ± 0.13 cm) were longer than that of ZH11 (2.05 ± 0.16 cm) (Figure 2c). Taken together, we concluded that overexpression of *OsF3H_04g_* results in increased susceptibility to RS105. OsF3H_03g_ negatively regulates the resistance to RS105 by inactivating SA in rice [4]. To test the effect of OsF3H_04g_ on SA, we then performed SA quantification on two *OsF3H_04g_* OE lines. The results showed that the SA content also decreased in the *OsF3H_04g_* OE lines (Figure 2d).

### 2.4. Editing EBE of the OsF3H_04g_ Promoter Compromised Tal2c-Mediated Susceptibility in Rice

**Figure 3:**
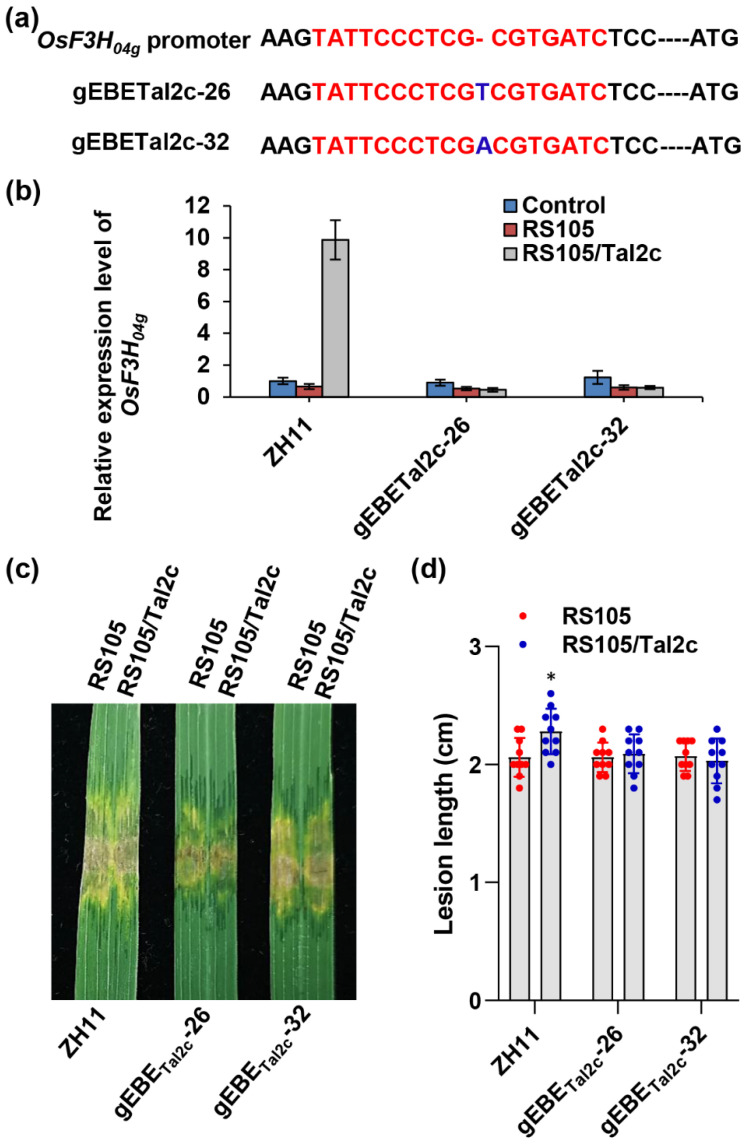
Gene editing of EBE in the *OsF3H_04g_* promoter decreases rice susceptibility to Xoc strain RS105/Tal2c. (**a**) Genotypes of the EBE gene editing lines (gEBETal2c-26 and gEBETal2c-32) in the *OsF3H_04g_* promoter. The sequences in red represent the Tal2c binding sites, and the bases in blue represent the insertion in the *OsF3H_04g_* promoter. (**b**) Relative expression level of *OsF3H_04g_* in ZH11, gEBETal2c-26 and gEBETal2c-32 lines at 4 dpi with RS105 and RS105/Tal2c. *OsACTIN* was used as an internal control. Data represent the means ± SD, *n* = 3. (**c**) Photograph of lesion expansion and (**d**) diagram of the lesion lengths in the ZH11, gEBETal2c-26 and gEBETal2c-32 lines at 14 dpi with RS105 and RS105/Tal2c. Data represent the means ± SD, *n* = 10. Asterisks represent significant differences between RS105 and RS105/Tal2c in ZH11 rice (* *p* ≤ 0.05, Student’s *t*-test).

Tal2c was predicted to bind EBE in the promoter of *OsF3H_04g_* to activate gene expression and promote infection. Thus, we designed guide RNA and performed gene editing of the EBE in the *OsF3H_04g_* promoter. Two individual lines were identified to homozygously insert a “T” and “A” base in lines gEBETal2c-26 and gEBETal2c-32, respectively (Figure 3a and Figure S3). We first measured the induction of *OsF3H_04g_* by inoculation with RS105 and RS105/Tal2c on the gEBETal2c-26 and gEBETal2c-32 lines. We observed that the induced expression of *OsF3H_04g_* by RS105/Tal2c was abolished in the two lines, gEBETal2c-26 and gEBETal2c-32, compared to the wild-type ZH11 (Figure 3b). In accordance with the deficient induction of *OsF3H_04g_*, the disease symptoms caused by RS105/Tal2c in gEBETal2c-26 and gEBETal2c-32 were milder than those in ZH11, while the disease symptoms caused by RS105 were similar to those in ZH11 and the two EBE gene-edited lines (Figure 3c). As shown in Figure 3d, similar lesion lengths caused by RS105 were observed in ZH11 (2.06 ± 0.16 cm), gEBETal2c-26 (2.06 ± 0.13 cm) and gEBETal2c-32 (2.07 ± 0.12 cm). However, the lesion lengths caused by RS105/Tal2c in gEBETal2c-26 (2.09 ± 0.17 cm) and gEBETal2c-32 (2.03 ± 0.19 cm) were shorter than that in ZH11 (2.28 ± 0.19 cm) and similar to those after inoculation with RS105. The above results indicated that the induction expression of *OsF3H_04g_* determined by EBE is required for the virulence of Tal2c.

### 2.5. Gene Editing of EBE in the OsF3H_03g_ Promoter Specifically Attenuates Rice Susceptibility to RS105/Tal2b

**Figure 4:**
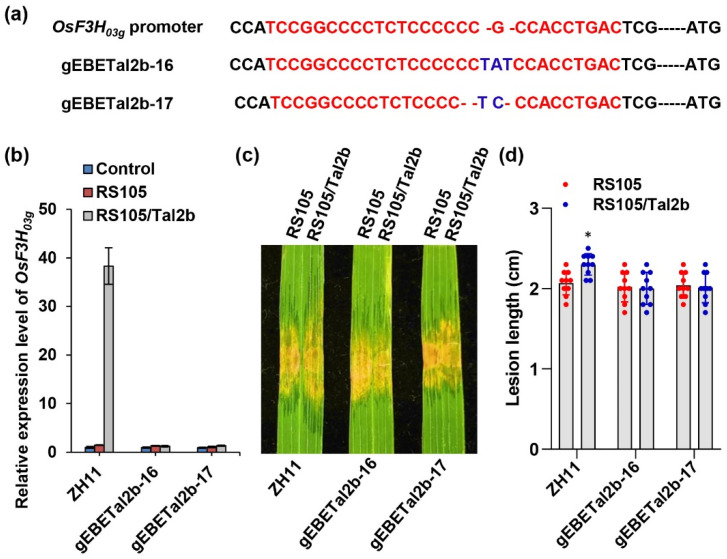
Gene editing of EBE in the *OsF3H_03g_* promoter reduces rice susceptibility to Xoc strain RS105/Tal2b. (**a**) Genotypes of the EBE gene editing lines (gEBETal2b-16 and gEBETal2b-17) in the *OsF3H_03g_* promoter. The sequences in red represent the Tal2b binding sites, and the bases in blue represent the substitution in the *OsF3H_03g_* promoter. (**b**) Relative expression level of *OsF3H_03g_* in the ZH11, gEBETal2b-16 and gEBETal2b-17 lines at 4 dpi with RS105 and RS105/Tal2b. *OsACTIN* was used as an internal control. Data represent the means ± SD, *n* = 3. (**c**) Photograph of lesion expansion and (**d**) diagram of lesion length in the ZH11, gEBETal2b-16 and gEBETal2b-17 lines at 14 dpi with RS105 and RS105/Tal2b. Data represent the means ± SD, *n* = 10. Asterisks represent significant differences between RS105 and RS105/Tal2b in ZH11 rice (* *p* ≤ 0.05, Student’s *t*-test).

We previously demonstrated that Tal2b could activate the expression of *OsF3H_03g_* and thus contribute to the virulence of Xoc HGA4 [4]. However, *OsF3H_03g_* was involved in negatively regulating rice immunity, and gene editing of the CDS of the gene resulted in broad resistance to Xoc and Xoo strains, regardless of whether they carried Tal2b [4]. To mine additional evidence to support the hypothesis that Tal2b redundantly functions with Tal2c, we also performed gene editing of EBE in the promoter of the Xoc susceptible gene *OsF3H_03g_* in ZH11. The two lines gEBETal2b-16 and gEBETal2b-17 were identified as homozygous substitutions of “TAT” to “G” and “TC” to “CCG” in the EBE region, respectively (Figure S4 and Figure 4a). RS105/Tal2b was abolished to induce the expression of *OsF3H_03g_* in both gEBETal2b-16 and gEBETal2b-17 (Figure 4b). After inoculation with RS105 or RS105/Tal2b on ZH11, gEBETal2b-16 and gEBETal2b-17, five out of six inoculations caused similar disease symptoms and lesion lengths, except RS105/Tal2b, which caused milder symptoms and longer lesion lengths on ZH11 compared with the five inoculations (Figure 4c,d). Similar to the above specific interaction between Tal2c and *OsF3H_04g_*, these results suggest that *OsF3H_03g_* is specifically required for Tal2b-mediated virulence.

### 2.6. Single Gene Editing of EBE in OsF3H_04g_ or OsF3H_03g_ Does Not Affect Resistance to Xoc HGA4

**Figure 5:**
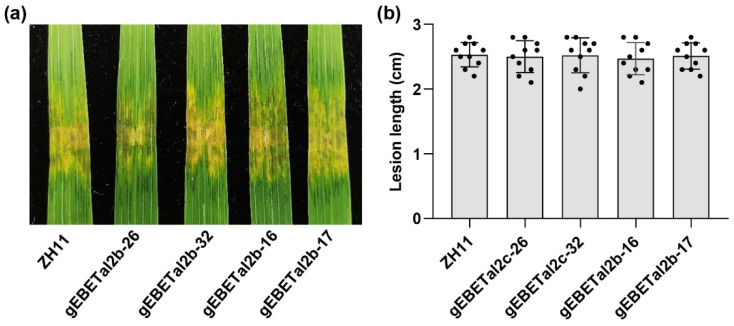
Single mutants of EBE in either the *OsF3H_04g_* or *OsF3H_03g_* promoter had no effect on rice susceptibility to Xoc strain HGA4. (**a**) Image of lesion expansion in ZH11, gEBETal2b-16, gEBETal2b-17, gEBETal2c-26 and gEBETal2c-32 lines at 14 dpi with HGA4. (**b**) Diagram of lesion lengths in ZH11, gEBETal2b-16, gEBETal2b-17, gEBETal2c-26 and gEBETal2c-32 lines at 14 dpi with HGA4. Data represent the means ± SD, *n* = 10.

We found that gene editing of EBE in *OsF3H_04g_* or *OsF3H_03g_* reduced rice susceptibility to Xoc strains RS105/Tal2c or RS105/Tal2b, respectively. However, neither of them had any significant effect on rice susceptibility to Xoc strain RS105, which contains Tal2b and Tal2c [4]. The hypothesis is that Tal2b and Tal2c are redundant pairs of TAL effectors; thus, gene editing of EBE in *OsF3H_04g_* or *OsF3H_03g_* would not affect resistance to Xoc strains containing Tal2b and Tal2c. We then performed a disease assay for inoculation of HGA4 after gene editing of EBE in *OsF3H_04g_* or *OsF3H_03g_* plants. The results showed that no significant difference in lesion expansion caused by HGA4 was observed in gEBETal2c-26 and gEBETal2c-32 compared with ZH11 or in gEBETal2b-16 and gEBETal2b-17 (Figure 5a). The lesion lengths in gEBETal2c-26 (2.5 ± 0.24 cm) and gEBETal2c-32 (2.52 ± 0.27 cm), gEBETal2b-16 (2.47 ± 0.25 cm) and gEBETal2b-17 (2.51 ± 0.20 cm) were similar to that in ZH11 (2.53 ± 0.19 cm) (Figure 5b). Thus, the results also supported that Tal2b and Tal2c contained in HGA4 may have redundant functions.

### 2.7. Comparative Analysis of the Transcriptome Profiles of the OsF3H_04g_ and OsF3H_03g_ Overexpression Lines

**Figure 6:**
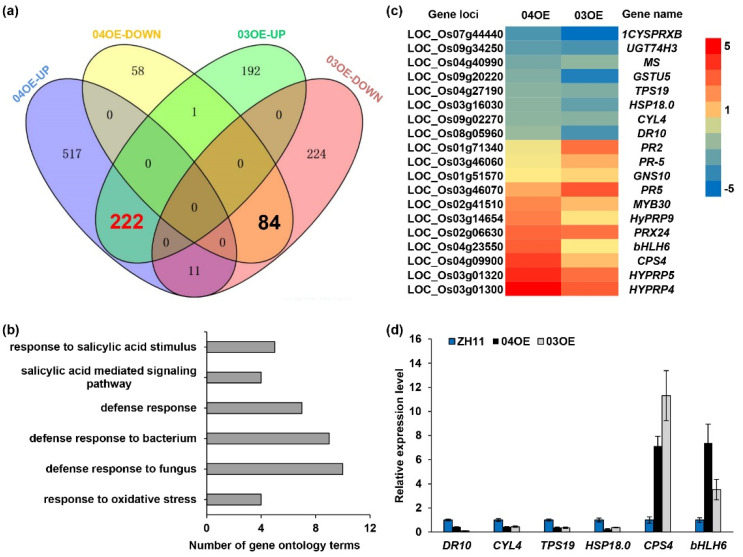
*OsF3H_04g_* negatively regulates the rice defense response to Xoc. (**a**) Venn diagram of the upregulated and downregulated genes in *OsF3H_04g_* (04OE-1) and *OsF3H_03g_* (03OE-2) OE lines. (**b**) Gene ontology terms of defense response-related pathways for the common differentially expressed genes (DEGs). The 222 upregulated and 84 downregulated genes in the *OsF3H_04g_* and *OsF3H_03g_* OE lines were analyzed and enriched. (**c**) Heatmap of common DEGs related to defense response, SA response and signaling pathway. (**d**) qRT–PCR analysis of the expression of SA-related genes and defense response genes in *OsF3H_04g_* and *OsF3H_03g_* OE lines. *OsACTIN* was used as an internal control. Data represent the means ± SD, *n* = 3.

Previously, we reported that overexpression of *OsF3H_03g_* could cause transcriptional reprogramming of numerous defense response genes using RNA-seq [4]. Simultaneously, RNA-seq of *OsF3H_04g_* OE plants was also performed for a parallel analysis of the differentially expressed genes (DEGs). As shown in Figure 6a, compared to ZH11, 750 upregulated and 143 downregulated DEGs were identified in the *OsF3H_04g_* OE line 04OE-1 (Table S1). We then performed a comparison of the DEGs between the line 04OE-1 and the *OsF3H_03g_* OE line 03OE-2. A total of 222 upregulated and 84 downregulated DEGs were identified as common DEGs, with 29.6% and 53.5% of the upregulated DEGs and 58.7% and 26.3% of downregulated DEGs sharing in the *OsF3H_04g_* and *OsF3H_03g_* OE lines, respectively (Figure 6a; Table S2). We further performed a gene ontology (GO) analysis for these 306 common DEGs. Six functional categories related to the defense response were enriched, with 5, 4, 7, 9 and 10 DEGs belonging to the response to salicylic acid stimulus, salicylic-acid-mediated signaling pathway, defense response and defense response to bacterium and fungus, respectively (Figure 6b). Otherwise, four DEGs belonged to the category of response to oxidative stress (Figure 6b). Overexpression of both *OsF3H_03g_* and *OsF3H_04g_* resulted in enhanced susceptibility and a reduction in SA levels in rice (Figure 2) [4]. We then analyzed the expression of SA-related and defense-related genes among the common DEGs (Figure 6c). SA-related genes, including the pathogen-induced defense-responsive gene *DR10* [39] and the cyclase-like gene *CYL4* [40], were downregulated in both rice OE lines. Additionally, the SA and JA signaling regulator gene *bHLH6* (LOC_Os04g23550), which negatively regulates rice immunity [41], was found to have enhanced expression in both *OsF3H_04g_* and *OsF3H_03g_* OE lines (Figure 6c,d). Among the GO functional categories of defense response, a small heat shock protein gene *HSP18.0-CI* [42,43] and a terpene synthase gene *OsTPS19* [44] have been identified as positively regulating resistance to phytopathogens in rice and were classified into the commonly downregulated DEGs in both *OsF3H_04g_* and *OsF3H_03g_* OE lines (Figure 6c,d). In addition, the syn-copalyl diphosphate synthase gene *OsCPS4* [45], which negatively regulates rice resistance to Xoo and *Magnaporthe grisea*, showed increased expression in both *OsF3H_04g_* and *OsF3H_03g_* OE lines (Figure 6c,d). Overall, the *OsF3H_04g_*- and *OsF3H_03g_*-overexpressing rice lines share a large number of common DEGs, suggesting that they may have similar biological functions in regulating rice immunity.

## 3. Discussion

**Figure 7:**
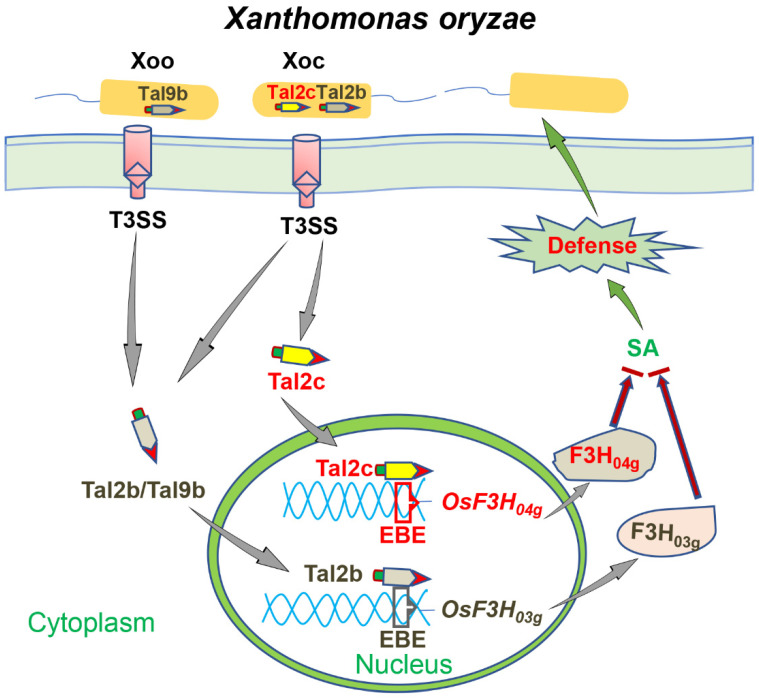
Working model of redundant Tal2b and Tal2c from *Xanthomonas oryzae* pv. *oryzicola*. Xoc delivers two TALEs, Tal2b and Tal2c, into rice cells using a type III secretion system (T3SS). Tal2b and Tal2c then enter the nucleus to directly bind to EBEs of the 2-oxoglutarate-dependent dioxygenase (2OGD) genes *OsF3H_03g_* and *OsF3H_04g_* promoters and activate gene expression, respectively. However, *Xanthomonas oryzae* pv. *oryzae* delivers only a Tal2b homologue, Tal9b, to directly bind to EBE of *OsF3H_03g_* promoter to promote infection. Finally, OsF3H_03g_ and OsF3H_04g_ work as similar functions in the negative regulation of defense and SA content in rice against Xoc.

In this work, we revealed that the TALE Tal2c acts as a virulence factor that shows a redundant function with previously reported Tal2b in most Xoc strains, including the highly virulent strain HGA4. The detailed interaction between the two TALEs and targets is illustrated in Figure 7, and these interactions are further described in the following main results. We identified that Tal2c binds to the EBE of the *OsF3H_04g_* promoter to promote infection in rice. Overexpression of *OsF3H_04g_* resulted in increased susceptibility, decreased SA accumulation and shared common DEGs with the *OsF3H_03g_*-OE line. The EBE editing lines of *OsF3H_03g_* and *OsF3H_04g_* specifically confer resistance to Tal2b- or Tal2c-containing strains. Overall, we significantly broadened the knowledge of TALE-mediated susceptibility to Xoc.

Most Xoc and Xoo strains carry dozens of TALEs that have been delivered into rice cells to support infection [46]. To date, several TALE-induced target genes have been identified that support the virulence of Xoo pathogens. For example, PthXo1 and *OsSWEET11* [47], PthXo2 and *OsSWEET13* [48], and *OsSWEET14* are targeted by four TALEs, including PthXo3, TalC, AvrXa7, Tal5 [48,49,50,51], PthXo6 and *OsTFX1* [52], PthXo7 and *OsTFIIAγ1* [52]. However, only a few Xoc TALEs and targets have been identified as promoting infection, such as Tal2g and *OsSULTR3;6* [13], Tal7 and Cyclin-D4-1 [16], and Tal2b and *OsF3H_03g_* [4]. A recent study also demonstrated that a TalI-deficient mutant compromised bacterial virulence and growth and acted as a virulence factor of Xoc without any predicted target in rice [53]. Previously, we identified that the highly virulent strain of Xoc HGA4 contains four expanded TALEs, namely, Tal2b, Tal2c, Tal2d and Tal2e [4]. Among them, Tal2b is a virulence factor targeting *OsF3H_03g_* after introduction into RS105, which does not contain the four TALEs [4]. Tal2c from Xoc (also known as TalBL) could activate the expression of putative target genes *OsF3H_04g_* (*OsDOX-2*) in *Nicotiana benthamiana* transient experiments [18]. In this study, we identified that Tal2c from Xoc HGA4 also acts as a virulence factor to promote infection in rice by introducing Tal2c into RS105 (Figure 1). Its target gene *OsF3H_04g_* was indeed a susceptibility gene (Figure 2). Thus, we provided a novel interaction that provides further insights into the virulence of TALE.

In previous studies, classical interactions between TALEs and targeted susceptibility genes have demonstrated that several TALEs in different Xoo strains target EBEs in the promoters of sugar transporter family genes *OsSWEET11*, *OsSWEET13* and *OsSWEET14* to promote infection in rice [47,49,51,54]. Due to the similar biological functions of their targets, these Xoo TALEs could be substituted with each other to defeat *xa13*-mediated resistance [49]. Recently, we reported that Tal2b from Xoc strain HGA4 targets the EBE of rice *OsF3H_03g_* as a virulence factor [4]. In this study, we found that another TALE, Tal2c, from HGA4 targets the EBE of rice *OsF3H_04g_* as a virulence factor. Both *OsF3H_03g_*- and *OsF3H_04g_*-overexpressing rice lines showed increased susceptibility to Xoc, decreased SA contents and altered the expression of defense response genes (Figure 2 and Figure 6). Thus, similar to Xoo, Tal2b and Tal2c from HGA4 may target similar functional enzymes to reduce the rice defense response to Xoc. Compared with the diverse TALEs in different Xoo strains, Tal2b and Tal2c clustered in most of the sequenced genomes of Xoc strains (Table 1), suggesting a redundant relationship. In addition, gene editing of the EBE in the *OsF3H_03g_* or *OsF3H_04g_* promoter correspondingly enhanced rice resistance to Xoc strains RS105/Tal2b or RS105/Tal2c but had no effect on rice resistance to Xoc strain HGA4, which carries both Tal2b and Tal2c (Figure 5). Thus, the Tal2b and Tal2c orthologues target functionally similar enzymes of the OsF3H family to redundantly regulate the rice defense response to Xoc. This could explain why a single mutant of Tal2b or Tal2c orthologue did not cause a significant loss of pathogenicity, as previously reported [13].

SA is an important hormone in the regulation of the plant defense response to a variety of phytopathogens [55]. To successfully infect plants, bacterial pathogens have evolved different tactics to block SA-mediated defense [22,46]. One of the tactics is disturbing the biosynthesis of SA. For example, HopI1 from *Pseudomonas syringae* pv. *maculicola* ES436 is translocated to the chloroplast and targets Hsp70 to suppress SA accumulation in the host [56,57]. The second tactic interferes with the SA signaling pathway. For example, *P*. *syringae* can generate coronatine to simulate JA signaling to antagonize SA signaling [58]. Otherwise, *P*. *syringae* secretes AvrPtoB to facilitate the ubiquitination and degradation of NPR1, the master regulator of SA signaling, to subvert plant defense [59]. The last tactic leads to the direct degradation of SA. There are many examples, such as *Ralstonia solanacearum*, which utilizes the Nag pathway to degrade SA and causes wilt disease in tomato [60], and *Candidatus* Liberibacter asiaticus, which secretes the hydroxylase SahA to degrade SA and causes huanglongbing (HLB) in citrus plants [61]. Recently, we reported that the Xoc strain HGA4 delivers a TALE Tal2b into rice to activate OsF3H_03g_ to decrease the SA content, which may occur through hydroxylation into 2,5-DHBA to promote infection in rice. Xoo also carries TALE Tal9b to target EBE in the *OsF3H_03g_* promoter to promote infection in rice [4,18]. In this study, we also found that Tal2c from HGA4 activates the similar-functioning enzyme OsF3H_04g_ to decrease SA content and increase a larger bacterial population (Figure 1 and Figure 2). However, both Tal2b and Tal2c are not necessarily required for maintaining complete pathogenicity because the strain RS105 could successfully infect rice in the absence of the two TALEs. Thus, our findings suggest a role to understand the mechanism by which Xoc and Xoo deliver TALEs to activate host SA metabolic enzymes to decrease the SA content. It would be better to maintain better living conditions for growth rather than interfering with key immunity in the host.

Previously, we found that OsF3H_03g_ plays a negative role in SA-mediated defense against Xoc [4]. Here, we also found that overexpression of *OsF3H_04g_* resulted in increased susceptibility to Xoc (Figure 2). These two rice genes were closely homologous to *AtDMR6,* which participates in SA metabolism in Arabidopsis [18,25]. These results were consistent with our findings that overexpression of *OsF3H_03g_* and *OsF3H_04g_* reduced the SA level and altered the expression of several SA-related genes in rice (Figure 2 and Figure 6). Thus, we concluded that *OsF3H_04g_* also plays a negative role in regulating SA-mediated defense against Xoc. We also observed the differences caused by overexpression of *OsF3H_03g_* and *OsF3H_04g_*. For instance, the SA response genes *OsbHLH187*, *OsWRKY45* and *OsNPR3* have been previously identified as exhibiting decreased and upregulated expression, respectively, in *OsF3H_03g_* OE and gene editing lines [4]. However, *OsbHLH187* and *OsWRKY45* were found to activate expression in the *OsF3H_04g_* OE line (Table S2). Moreover, a severe reduction in SA was identified in *OsF3H_03g_* OE lines [4], while a mild decrease in SA was observed in *OsF3H_04g_* OE lines (Figure 2d). These results implied that OsF3H_04g_ may have other functions in the regulation of the rice defense response in addition to participating in SA-related defense against Xoc. Heterogeneous expression of *OsF3H_03g_* (LOC_Os03g03034) or *OsF3H_04g_* (LOC_Os04g49194) in the Arabidopsis *AtF3H* gene-deficient mutant *tt6* did not recover anthocyanin accumulation [62]. Overexpression of *OsF3H_03g_* in rice could improve flavonoid and anthocyanin contents and result in increased resistance to rice brown planthoppers [31]. Thus, whether *OsF3H_04g_* plays a role in the regulation of flavonoid and anthocyanin accumulation in rice needs further study.

Previously, we found that gene editing in the CDS region caused a deficient function of OsF3H_03g_ and broad resistance to BB and BLS. However, constitutive activation of resistance results in agronomic trait costs [4]. Recent studies have shown that gene editing of the EBE in the promoter of the Tal2g target *OsSULTR3;6* enhances rice resistance to different Xoc strains without any observed changes in most agronomic traits [36,37]. In this study, we found that gene editing of EBEs in the *OsF3H_03g_* or *OsF3H_04g_* promoter conferred rice with resistance to Xoc strains RS105/Tal2b and RS105/Tal2c, respectively, but did not enhance rice resistance to the wild-type Xoc strains RS105 and HGA4 (Figure 3 and Figure 4). Additional Xoc strains carry both Tal2b and Tal2c orthologues (Table 1). We proposed that double gene editing of both EBEs in the *OsF3H_03g_* and *OsF3H_04g_* promoters and the pyramiding of gene editing of all EBEs in *OsSULTR3;6* could greatly improve rice production and provide broad-spectrum resistance to Xoc.

## 4. Materials and Methods

### 4.1. Plant Materials and Growth Condition

Rice ZH11 (*Oryza sativa* L. ssp. *japonica*) variety which is moderately susceptible to BLS was used for gene expression pattern and transgenic manipulation. The rice seeds were grown in the greenhouse under the temperature of 28 ± 2 °C, humidity of 85% to 100% and photoperiod of 12 h. *Nicotiana benthamiana* was used for the transient expression, and it was grown under the temperature of 22 ± 2 °C, humidity of 40% to 50% and photoperiod of 16 h in growth chamber.

### 4.2. Transient Expression

The 3282 bp DNA fragment containing coding sequence (CDS) of *Tal2c* gene was amplified from HGA4 genomic DNA, and subsequently cloned into the pCXSN-MYC vector to construct pSN-Tal2c [63]. The 1000 bp promoter fragment upstream initiation site of *OsF3H_04g_* gene was cloned into pCXGFP-P vector to generate 04gPRO-GFP, and the DNA fragment which deleted the EBE was generated by overlapping PCR and then cloned into pCXGFP-P vector to generate 04gPROΔEBE-GFP. Above plasmids were transformed into *Agrobacterium tumefaciens* GV3101 separately. As previously reported [64], the *A*. *tumefaciens* carried-different vectors were co-infiltrated into *N*. *benthamiana* cells, the GFP signal was observed at 3 days after infiltration using fluorescence microscope (Nikon, Tokyo, Japan).

### 4.3. Construction of the RS105/Tal2c Strain and Bacterial Inoculation

The *Tal2c* gene, including the 129 bp promoter and 3282 bp CDS, was amplified from HGA4 genomic DNA with special primers (Table S3) and then cloned into the pVSP61 vector to generate pVSP61-Tal2c. The pVSP61-Tal2c vector was transformed into RS105 competent cells according to previous studies [4,65]. The Xoc strains RS105, HGA4 and RS105/Tal2c were incubated on PSA plates for 2 to 3 days. The bacteria were eluted from the plates, and the OD600 was adjusted to 0.5 with sterile water separately. The bacterial suspensions were infiltrated into 8-week-old rice leaves using a 2 mL no-needle syringe. The bacterial growth curve and relative bacterial biomass were determined at 4 and 8 dpi with RS105 or RS105/Tal2c according to a previous study [4].

### 4.4. RNA Manipulation and RNA-seq

Eight-week-old ZH11 rice leaves were inoculated with Xoc strains and then collected at 4 dpi for RNA extraction using Monzol^®^ Reagent (Monad, Wuhan, China). First strand cDNA was synthesized using MonScript™ RTIII All-in-One Mix with dsDNase (Monad, Wuhan, China). cDNA was used for qRT–PCR with MonAmp™ SYBR^®^ Green qPCR Mix (Monad, Wuhan, China) in a qTOWER^3^G Real-Time PCR System (Analytikjena, Jena, Germany). The primers used for qRT–PCR are listed in Table S3.

As previously reported [4], the 04gOE-1 was grown for 8 weeks along with ZH11, and leaves were collected for RNA extraction using a plant RNA kit (OMEGA Bio-Tek, Norcross, GA, USA). Three different repeats of RNA were used to construct libraries and perform RNA-seq by BGISEQ-500 (Beijing Genomics Institution, Shenzhen, China) according to previous reports [42,43,65]. The accession number (PRJNA781784) was obtained from the NCBI Sequence Read Archive (SRA) after submitting the raw sequence reads. The significant DEGs were screened by absolute log2-ratio values ≥1 in 04gOE vs. ZH11 (simultaneously sequenced with 04gOE and 03gOE which previously uploaded files as PRJNA730674) and *p* ≤ 0.05. The DEGs in *OsF3H_03g_* OE line were obtained from NCBI SRA with accession number PRJNA730674 [4]. The common DEGs were identified by Venny 2.1 (https://bioinfogp.cnb.csic.es/tools/venny/index.html, accessed on 8 September 2021), and gene ontology (GO) analysis was performed by PlantRegMap (http://plantregmap.gao-lab.org/go.php, accessed on 26 September 2021) and Oryzabase (https://shigen.nig.ac.jp/rice/oryzabase/gene/advanced/search, accessed on 26 September 2021).

### 4.5. Vector Construction and Rice Transformation for OsF3H_04g_ Overexpression and EBE Gene Editing Plasmids

To construct the overexpression rice, the 1023 bp fragment of *OsF3H_04g_* was amplified from ZH11 rice cDNA and cloned into the pCXUN-MYC vector to generate pUN-F3H04g. Gene editing of the rice genome was implemented by the CRISPR–Cas9 system according to previous reports [65,66]. The CRISPR–Cas9-mediated gene editing of gEBETal2c (5′-TATTCCCTCGCGTGATC-3′) in the *OsF3H_04g_* promoter and gEBETal2b (5′-TCCGGCCCCTCTCCCCCCGCCACCTGAC-3′) in the *OsF3H_03g_* promoter was designed with the U3-gRNA targets 5′-GCCGGCCGGAGATCACGCGA-3′ and 5′-AAGTCGAGTCAGGTGGCGGG-3′, respectively. The above gRNA targets were cloned into the pYLCRISPR/Cas9-MH vector [66]. The above vectors were transformed into *A. tumefaciens* EHA105 and then subjected to *A*. *tumefaciens*-mediated callus transformation in ZH11 rice.

### 4.6. Hormone Treatment

The leaves of wild-type ZH11 and *OsF3H_04g_* overexpression rice lines were collected for hormone quantitative analysis. In total, 100 mg of leaf tissue of ZH11 and *OsF3H_04g_* overexpression rice lines was ground in liquid nitrogen and then used for hormone extraction according to the previous report [4,67].

## 5. Conclusions

In this study, we validated the virulence function of Tal2c by introducing it into RS105. It activated the expression of *OsF3H0_4g_* to reduce SA content as well as to increase susceptibility in rice. We compared the interaction of Tal2c and *OsF3H_04g_* with previously identified Tal2b and *OsF3H_03g_* using transcriptome profiling and EBE gene editing. Intriguingly, our data suggest that the pair of Tal2b and Tal2c acts as redundant TALEs by specifically hijacking homologues of rice 2OGD gene. Thus, our research uncovers a virulence mechanism of Xoc and provides guidance for breeding BLS disease-resistant rice.

## Figures and Tables

**Table 1:** The Tal2b and Tal2c orthologs appeared to be tandem pairs in most Xoc strains.

Xoc Strains	Tal2b Ortholog	Tal2c Ortholog	Area of Isolation
HGA4	+	+	China
RS105	−	−	China
L8	+	+	China
B8-12	+	+	China
GX01	+	+	China
0–9	+	+	China
BLS256	+	+	Philippines
BLS279	+	+	Philippines
CFBP2286	+	+	Malaysia
CFBP7331/MAI10	+	+	Mali
CFBP7341/BAI5	+	+	Burkina
CFBP2286/BAI11	+	+	Burkina
BXOR1	+	+	India

## Data Availability

Sequence data from this study can be found in the Rice Genome Annotation Project website (http://rice.plantbiology.msu.edu/, accessed on 25 November 2021), NCBI (https://www.ncbi.nlm.nih.gov/, accessed on 25 November 2021) and the *Xanthomonas* Resource (http://www.xanthomonas.org/t3e.html, accessed on 25 November 2021) under the following accession number: *OsF3H_04g_* (LOC_Os04g49494), Tal2c of BLS256 (XOC_1570) and HGA4 (CP064794). Raw sequence reads of transcriptome sequencing for ZH11 and *OsF3H_03g_* transgenic rice were obtained from the NCBI Sequence Read Archive (SRA) with accession number of PRJNA730674. Raw sequence reads of transcriptome sequencing for *OsF3H_04g_* transgenic rice were performed in this study and uploaded to SRA to achieve the accession number PRJNA781784.

## Supplementary Materials

The following are available online at https://www.mdpi.com/article/10.3390/ijms222413628/s1.
