# Supplementary material for: Tal2c Activates the Expression of OsF3H04g to Promote Infection as a Redundant TALE of Tal2b in Xanthomonas oryzae pv. oryzicola
Source: Int J Mol Sci. 2021 Dec 20;22(24):13628. doi: 10.3390/ijms222413628 (PMC8707247; doi:10.3390/ijms222413628)
Supplement: Supplementary file 1 [file ijms-22-13628-s001.zip › Supplementary Figure S1-S4, Table S3.pdf]

Supplement figures

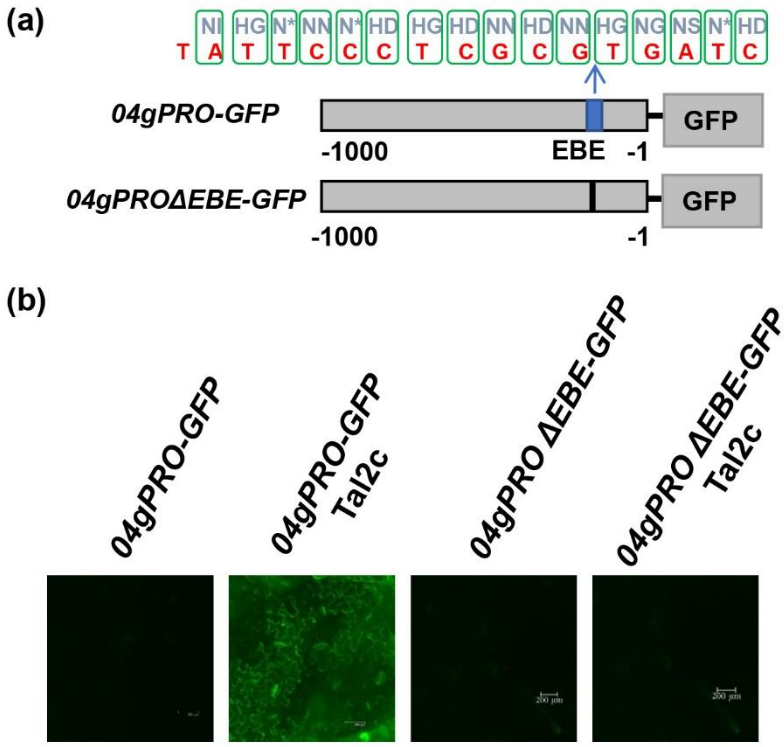

Figure S1. Tal2c directly targets the EBE in the promoter of *OsF3H$_{04g}$*. (A) Schematic diagram of the 1000 bp *OsF3H$_{04g}$* promoter and putative TAL effector binding element (EBE)-deleted promoter upstream of the translation initiation site fused with a green fluorescent protein (GFP) reporter. (B) Co-expression of Tal2c and GFP reporter derived under the *OsF3H$_{04g}$* promoter or EBE deletion promoter in *Nicotiana benthamiana*. The *OsF3H$_{04g}$* promoter- or EBE-deleted promoter-derived GFP reporter was individually expressed as a control. GFP fluorescence was scanned by laser confocal microscopy. Scale bars, 100 µm.

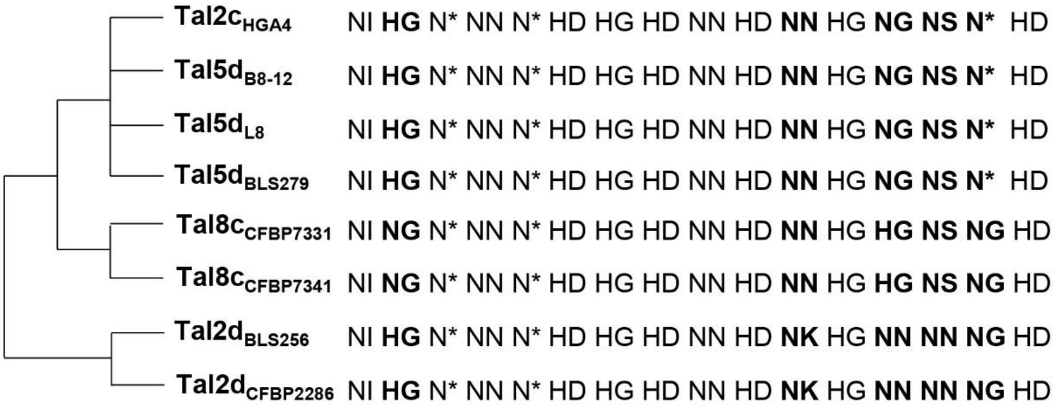

Figure S2. Alignment of RVD sequences of Tal2c orthologs from different Xoc strains. The RVD sequences of the Tal2c orthologs from HGA4, B8-12, L8, BLS279, BLS256, CFBP2286, CFBP7331, and CFBP7341 were aligned. The RVDs sequences labeled in dark indicate variations.

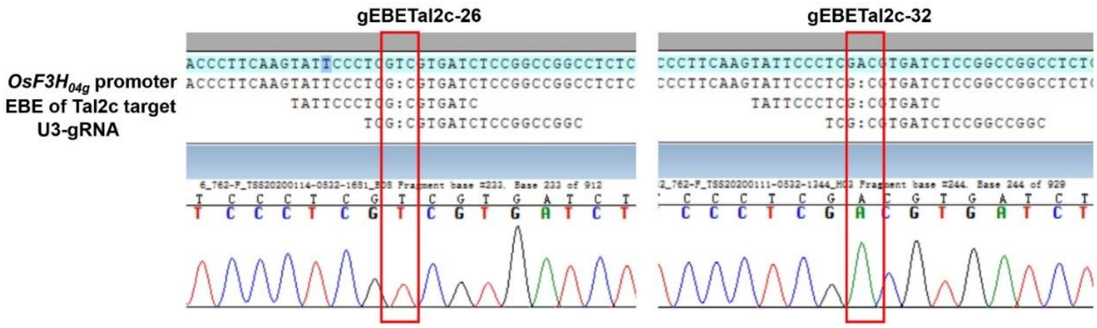

Figure S3. The blast of *OsF3H04g* promoter sequences of EBE gene editing rice lines.

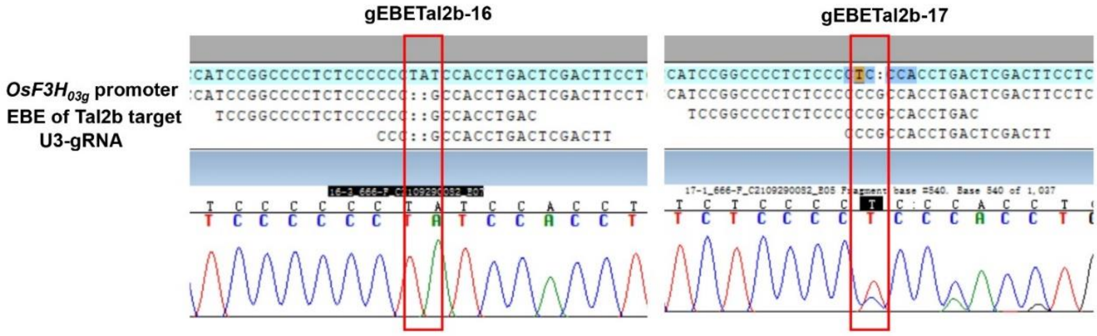

Figure S4. The blast of $\textit{OsF3H}_{\textit{03g}}$ promoter sequences of EBE gene editing rice lines.

Table S3. The detailed list of primers used in this study.

Primer name	Gene	Forward primer (5'-3')	Reverse primer (5'-3')	Application
Tal2c-F/R	Tal2c	atgaagcttGCTCAGTCGGGGGACTATCTGAGCGAC	atggtcgacTCACTGAGGCAATAGCTCCATCAACCATGCG	Amplification
Tal2b-F2/R2	Tal2c	GGATCCCATTCGTCCGCGCAGGC	TCAAGCGTAATCTGGAACATCGTATGGGTACTGAGGC	Amplification
04gPRO-F/R	Os04g49194	GGCTAGTGGTTGTGAGCCCCAAC	GGGAGACGAAGCGCGAGGT	Amplification
04gPRO-ΔEBE-F/R	Os04g49194	AGAGGCCGGCCGGAGATCCTTGAAGGGTGAATGCGCTACGCG	CGCGTAGCGCATTCACCCTTCAAGGATCTCCGGCCGGCCTCT	Amplification
F3H04g-OE-F/R	Os04g49194	GGCAACGACGCAGTTGCTGTC	CTACTGGCCTTTGAAGAGCTCTAGG	Amplification
EBETal2c-F/R	Os04g49194	ggcaGCCGGCCGGAGATCACGCGA	aaacTCGCGTGATCTCCGGCCGGC	U3-gRNA target
EBETal2b-F/R	Os03g03034	ggcaAAGTCGAGTCAGGTGGCGGG	aaacCCCGCCACCTGACTCGACTT	U3-gRNA target
F3H04g-GD-F/R	Os04g49194	AGCATTGGTGATGCCTTTAGG	TGTGTACTGTCTCCTTGCGGAC	Amplification
F3H03g-GD-F/R	Os03g03034	GCTCGATGTGGAGGGAGACTG	TGGAGCAGTGATGACTGACTTGC	Amplification
HPT-F/R	ABY65250	GATCGTTATGTTTATCGGCACTTTG	GTACTTCTACACAGCCATCGGTCCA	Positive identification
OsACTIN-qRT-F/R	Os03g50885	CAGGCCGTCCTCTCTCTGTA	ACCGATACTTGGACAG	qRT-PCR
Xoc0105-F/R	Xoc0105	CAGTTGCGTGCGTAGTTCAG	ACAGCAGTTCGATGCCTACA	qRT-PCR
F3H04g-qRT-F/R	Os04g49194	GTCACCAACCACGGGATAGC	AGTCTGATTTTCTTGGATGGCTC	qRT-PCR
F3H03g-qRT-F/R	Os03g03034	GAACGCCCTCACCATCCTC	GCTCTTGTATCTTCCGTTGCTTAG	qRT-PCR
DR10-qRT-F/R	Os08g05960	TCATCAAGCTGATTTCATCAGACA	CGTACTTGTAGAACGCCATGGA	qRT-PCR
CYL4-qRT-F/R	Os09g02270	CCACGTCTTCGACCACTAC	CTAGGAACATCGACCAACAA	qRT-PCR
TPS19-qRT-F/R	Os04g27190	GAGTGCTATATCAATGAGCAC	ACAGATTCCCAACATTTCCC	qRT-PCR
HSP18.0-qRT-F/R	Os03g16030	GGTGGAGAGCTTCGATTCGA	GGACCAGATTTGACGCTTTTATTT	qRT-PCR
CPS4-qRT-F/R	Os04g09900	CCCCACCTCCACTACCAATTC	AAACCCCGTCTCAGGAAAAAC	qRT-PCR
bHLH6-qRT-F/R	Os04g23550	ATTTTTGCGACACCCCACTA	GCTGGACCACCTTATTATTCATC	qRT-PCR
